# Supplementary material for: Differential Proteomic Analysis of Extracellular Vesicles Produced by Granulicatella adiacens in Biofilm vs. Planktonic Lifestyle
Source: Dent J (Basel). 2025 Nov 26;13(12):557. doi: 10.3390/dj13120557 (PMC12732219; doi:10.3390/dj13120557)
Supplement: Supplementary file 1 [file dentistry-13-00557-s001.zip › dentistry-3908660-supplementary.docx]

**Supplementary Figures**

A


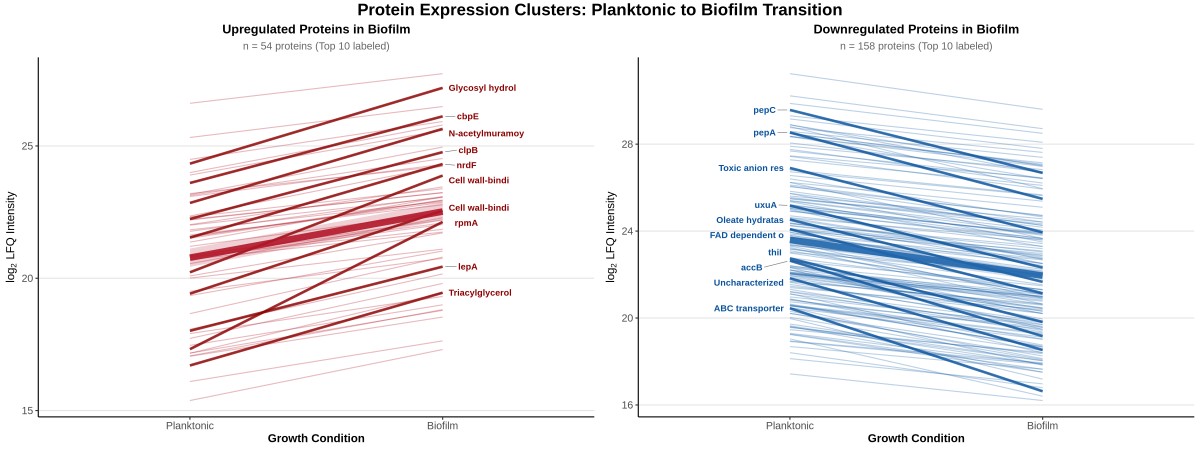


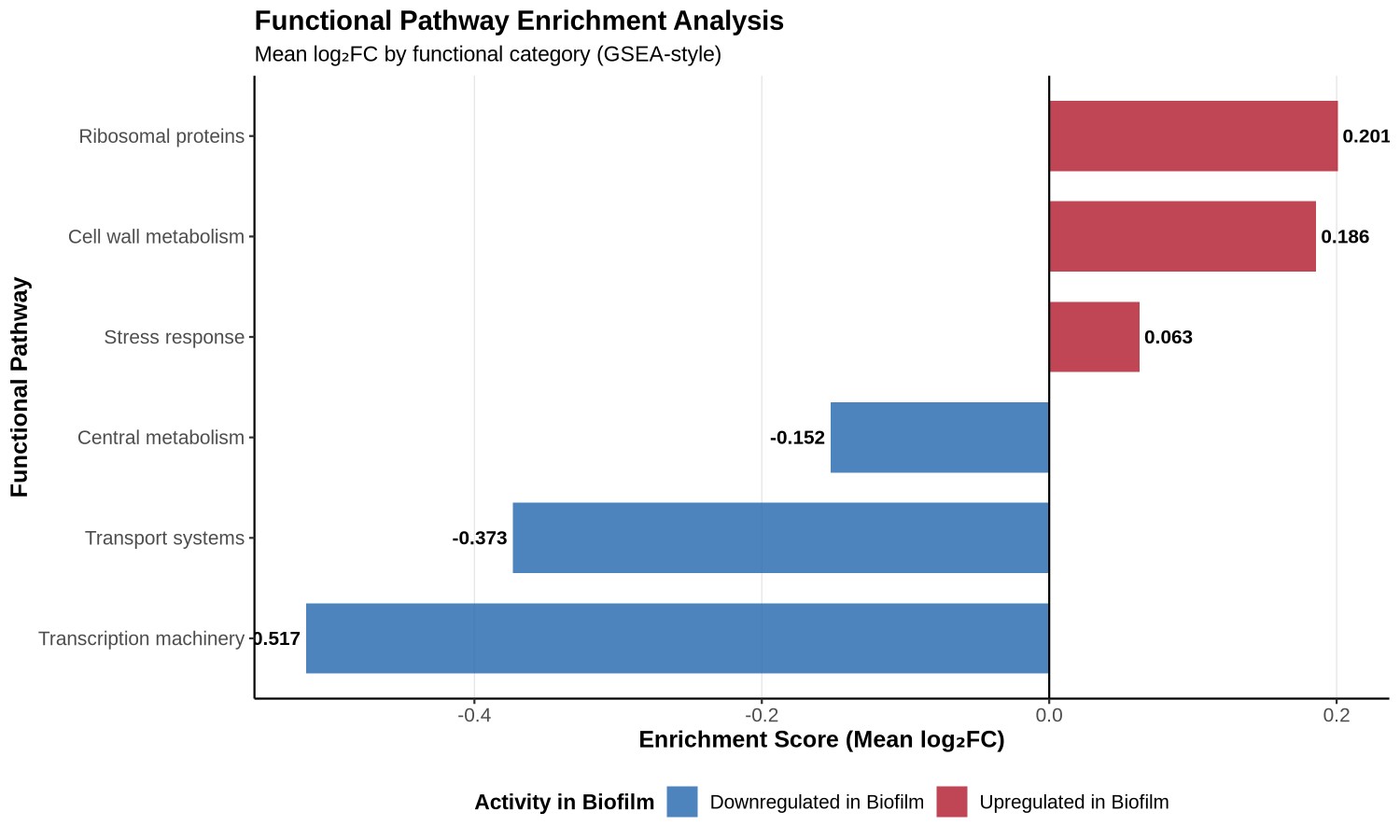


B

**Figure S1. Global proteomic landscape of *G. adiacens* EVs from biofilm and planktonic cultures.** This figure presents a global differential proteomic analysis, highlighting the distinct molecular signatures of extracellular vesicles (EVs) from *G. adiacens* grown in biofilm versus planktonic states. (A) Line plots showing log₂-transformed intensity profiles for proteins significantly upregulated in biofilm (a, red lines) and downregulated in biofilm (i.e., upregulated in planktonic; B, blue lines) relative to planktonic conditions, visualizing the consistent trend of differential expression across biological replicates. (B) Functional pathway enrichment analysis presented as a Gene Set Enrichment Analysis (GSEA) bar chart. The plot shows the mean log₂ fold-change (log₂FC) of proteins within key functional pathways. Red bars indicate pathways with activity upregulated in biofilm EVs (for example, Cell wall metabolism, Enrichment Score = 0.106; Stress response, Enrichment Score = 0.083), while blue bars indicate pathways with activity downregulated in biofilm EVs (for example, Transport systems, Enrichment Score = -0.373; Transcription machinery, Enrichment Score = -0.517).


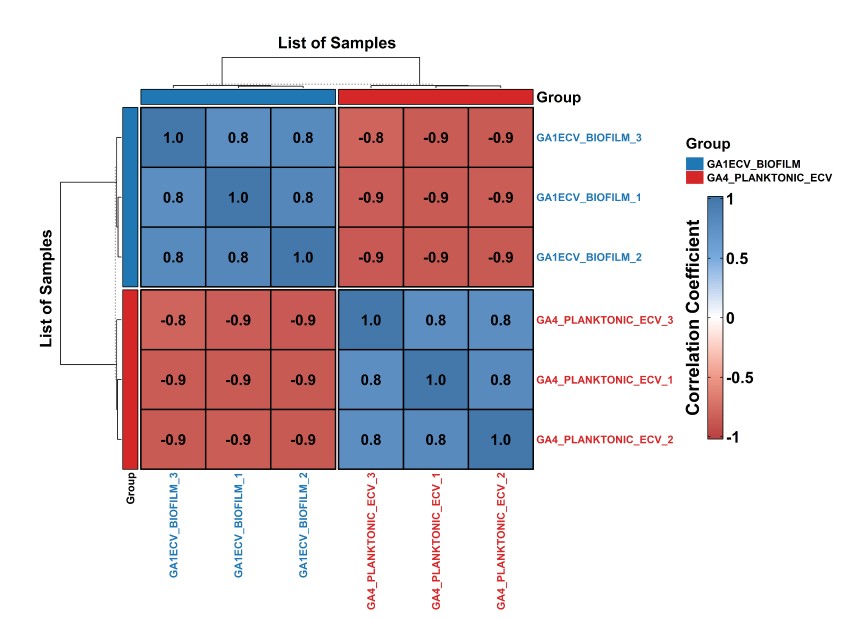

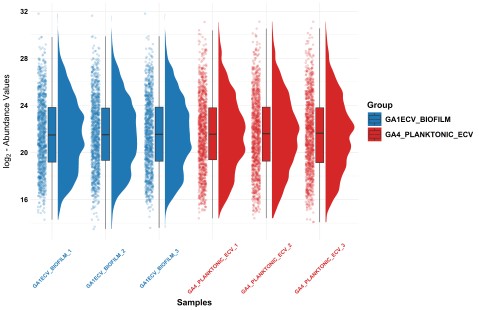


A

B

**Figure S2**. **Quality assessment of differentially expressed EV proteins.** A. Sample-to-sample correlation matrix visualized as a heatmap of Pearson correlation coefficients calculated from the entire quantified proteome. The high intra-group correlation (red squares, values approaching 1.0) and low inter-group correlation (blue squares) serve as a key quality control metric, demonstrating high biological reproducibility within conditions and distinct profiles between conditions. B. Violin plots showing the distribution of log₂-transformed protein abundances for each biological replicate, illustrating the overall consistency of data distributions across all samples following normalization and imputation.


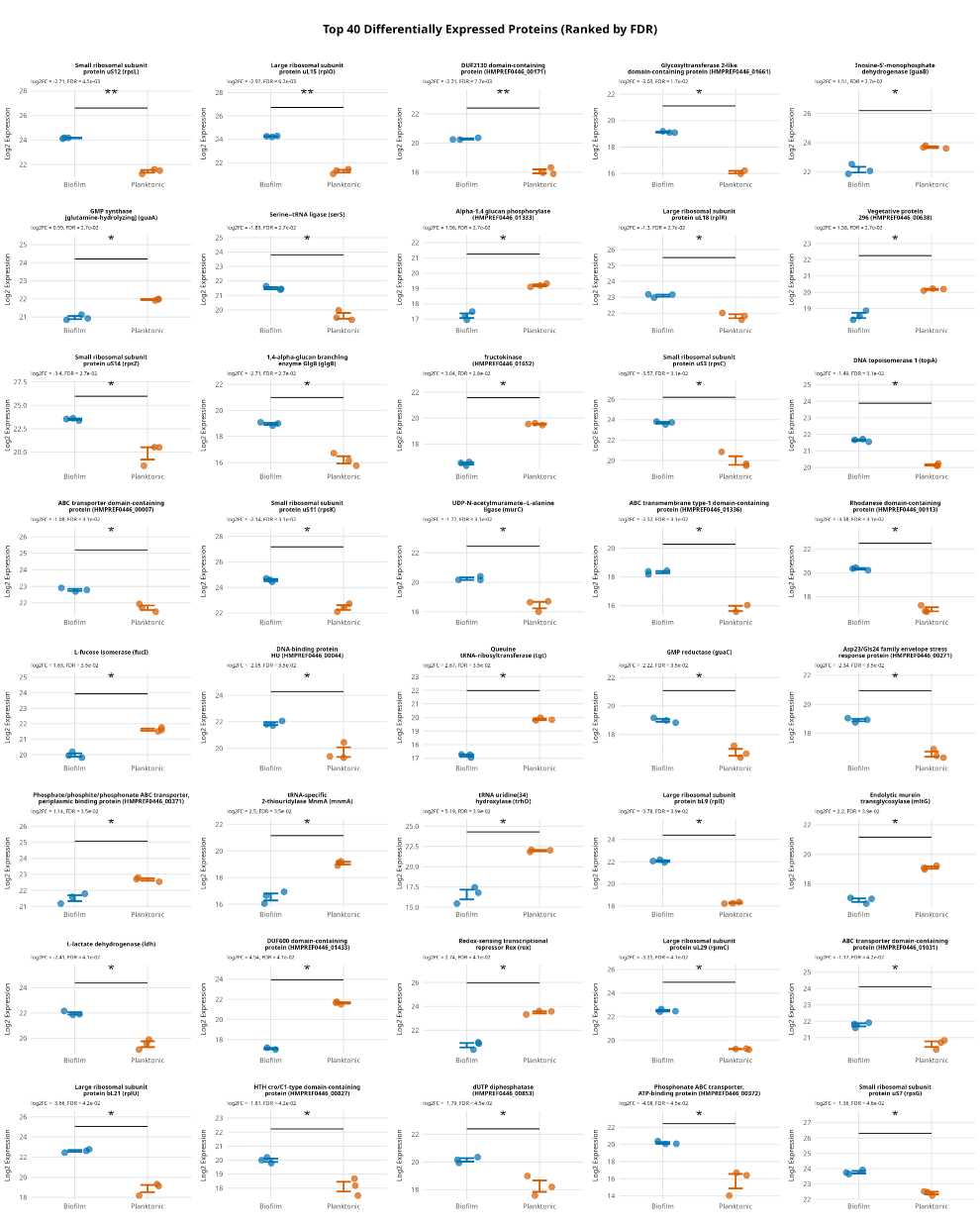


**Figure S3**. **Expression levels of proteins:** Scatter plots showing the expression levels of representative, individual, significantly differentially expressed proteins. Each plot displays the abundance for each of the three replicates in the biofilm (blue) and planktonic (orange) conditions, with statistical significance indicated (p < 0.05, p<0.01*).
